# Marginal Bone Loss in Internal Conical Connection Implants Placed at the Crestal and Subcrestal Levels before Prosthetic Loading: A Randomized Clinical Study

**DOI:** 10.3390/ma15103729

**Published:** 2022-05-23

**Authors:** Natalia Palacios-Garzón, Elisabeth Mauri-Obradors, Raúl Ayuso-Montero, Eugenio Velasco-Ortega, José María Anglada-Cantarell, José López-López

**Affiliations:** 1 Faculty of Medicine and Health Sciences (Dentistry), University of Barcelona, 08907 L’Hospitalet de Llobregat, Spain; npalaciosgarzon@gmail.com (N.P.-G.); lismauri3@gmail.com (E.M.-O.); 2 Oral Health and Masticatory System Group, Bellvitge Biomedical Research Institute IDIBELL, University of Barcelona, 08907 Barcelona, Spain; raulayuso@ub.edu (R.A.-M.); jmanglada@ub.edu (J.M.A.-C.); 3 Department of Odontostomatology, Faculty of Dentistry, University of Seville, 41009 Seville, Spain; evelasco@us.es; 4 Service of the Medical-Surgical Area of Dentistry Hospital, University of Barcelona, 08907 Barcelona, Spain

**Keywords:** external connection, internal connection, peri-implant bone loss, bone remodeling, preload prosthesis

## Abstract

The vertical position concerning the bone in which the implants are placed has been related as one of the factors causing marginal bone loss. The objective of this study was to evaluate the bone loss that occurs before prosthetic loading around tapered internal connection (CIC) implants placed at the crestal (C) and subcrestal (S) levels. Method: A randomized clinical trial (RCT) was carried out, with a sample size of 62 implants placed in 27 patients who underwent radiological controls on the day of placement, at one month, and at 4 months, and stability was measured by resonance frequency analysis (RFA) on three occasions. Results: Bone loss in implants C and S from the time of placement (T0) and the month after (T1) was not significant (*p* = 0.54) (C = 0.19 mm and S = 0.15 mm). The difference between one month (T1) and four months (T2) (C = 0.17 mm and S = 0.22 mm) was not significant either (*p* = 0.26). The difference between the day of placement (T0) and the third and last measurement (T2) was almost null (*p* = 0.94) (C = 0.35 mm and S = 0.36). The overall success rate of the implants was 97.8%. The stability of the implants measured with RFA went from 70.60 (T0) to 73.16 (T1) and 74.52 (T2). Conclusions: No significant differences were found in the bone loss for implants placed at the C and S levels. The millimeters of bone loss detected in both vertical positions did not have a significant impact on the stability of the implants.

## 1. Introduction

One of the challenges of current research in implantology is to reduce the minimal bone loss that occurs in the most coronal area of the implant, and that is more accentuated during the first year [1]. 

According to the standards of optimal conditions that implants must meet, it is said that they must be surrounded by a specific quantity (minimum 2mm)of quality bone at the morphological level [2]. This parameter is difficult to comply with within the vestibular area; here, we find a type of bone that, when there are teeth, can measure 1 mm or less. The vestibular bone is especially sensitive to the changes produced by the surgical act that occurs when the extraction and placement of the implant are performed [3]. Taking into account the particularity of this bone, a subcrestal implant placement approach could be favorable if we take into account that the preservation of this crestal bone can provide us with the stability of our soft tissues, a crucial element for the success and aesthetics of our treatment [4].

It has been suggested that a subcrestal implant placement may favor marginal bone preservation [5]. This concept is not current; it dates back to the 1960s, when the promoter of implantology, Dr. Branermark, already recommended it as one of his criteria for the success of implants, stating that burying the implant was necessary so that it would not be exposed after the bone remodeling process occurs [6]. 

This fact continues to be much discussed today. According to the latest studies collected in a systematic review and meta-analysis where only this aspect was assessed as the cause of bone loss, we can see that the literature is not clear enough. On the one hand, some authors defend the subcrestal placement of the implants, arguing the good results of its use. Especially in aesthetic areas, these studies affirm that the fact of deepening the implant would help to have a better emergence profile [7], preventing the implant surface from being exposed in the long term, thus reducing the probability of suffering from peri-implantitis [8,9]. On the other hand, the authors who defend a crestal position of the implant obtained evidence that demonstrates an increase in bone loss when the implant is buried. This loss could be due to bacterial colonization in the junction area between the implant and the abutment which, being deep, could be ideal for the proliferation of anaerobic bacteria [10,11,12]. 

The present work focuses on analyzing the bone loss that occurs due to the vertical position concerning the bone in which the implant is placed. However, many factors cause this loss; among these, we find the type of connection [13], surface [14], bone remodeling [15], occlusal load [16], mechanical imbalances [17], and bacterial infiltration [18]. Bone loss can be due to several factors that can be present at the same time, and it is difficult to isolate just one of them to assess it more precisely. Taking these limitations into account, in this clinical study, an attempt was made to control all the parameters that could affect bone loss as much as possible. Both positions of the implants had the same connection, macro design, and type of surface in the body of the implant; the closure abutment was placed in all of them, and the second phase was performed at a later time. Bone loss was analyzed before prosthetic loading.

## 2. Materials and Methods

### 2.1. Study Population

A randomized clinical study was carried out at the University of Barcelona Dental Hospital. This was approved by the ethics committee CEIC HOUB (Comitè d’Etica i Investigació Clínica de l’Hospital Odontològic Universitat de Barcelona) on 5 May 2017, with approval code 648. The criteria of the CONSORT guidelines for conducting clinical trials were taken into account, and they were included in the clinical trials database ClinicalTrials.gov ID: NCT03232372. 

The patients had to present total or partial edentulism in the mandible and/or maxilla, with at least 2 dental absences in the same maxilla. It was established that the implants should be placed in areas where there was adequate bone volume so that no threads would be exposed during surgery. In this way, the use of biomaterials was avoided in an attempt to reduce bias. In all cases, a diagnostic CBCT was performed to ensure that the area where the implants were to be placed had sufficient bone volume. It was established that a minimum height of 8 mm and a bone width >7 mm were allowed, except for the anterior sector, where, due to bone limitations [17], a width of 5–6 mm was allowed. Teeth should have been extracted at least 4–6 months before implant placement. Patients with serious systemic diseases, such as recent heart attack, coagulation disorders, cancer, uncontrolled diabetes, psychiatric contraindications, and active infection, as well as pregnant or lactating women, were excluded. Patients with pharmacological treatments capable of affecting bone healing (Bisphosphonates) and gingival health, such as some anticonvulsants (Phenytoin), immunosuppressants (Cyclosporin A), and calcium channel blockers (Nifedipine, Verapamil, and Diltiazem), were also excluded.

All patients received instructions to improve their oral hygiene prior to the implants’ placement. An intraoral examination took place where previous dental treatments, dental absences, plaque index, probing, mesial and distal contact of the area to be implanted, and type of teeth or opposing prosthesis were recorded. 

### 2.2. Study Design and Randomization

Before the surgery, the researcher (NPG) performed the randomization and assignment of the implants into two groups: A, the subcrestal group; and B, the crestal group. With this purpose, an application generating random numbers was used (Random Numbers—Version 4.2, 2015). Taking into account research that deals with the design and analysis of studies of split mouths, it was decided that patients had to have at least two edentulous spaces in the same jaw [19]. Thus, on the basis that the ossification of the mandible and the maxilla are different from each other [20], this could reduce the bias when comparing bone loss. 

In order to calculate the sample size required to test the hypothesis on the difference in bone loss of implants placed at the crestal and subcrestal levels, the following formula was used:

$$n=\frac{\;\;\;2{\left(Z_{\alpha }+Z_{\beta }\right)}^{2}\times S^{2}}{d^{2}\;}$$

where *n* is the subjects in each of the two groups (crestal vs. subcrestal); *Z_α_* is the *Z*-value correspondent to the accepted possible risk of mistakenly rejecting a true null hypothesis; *Z_β_* is the *Z*-value correspondent to the accepted possible risk of failing to reject a false null hypothesis; *S* is the standard deviation in mm of bone loss in implants placed at crestal and subcrestal levels; and *d* is the minimum desired value in mm of the difference in bone loss between the two groups.

After carefully reviewing the related literature [9,21,22], it was found that the average standard deviation of bone loss for implants placed at crestal level was approximately 0.35 mm, while, to obtain a significant difference with implant placed at subcrestal level, it would be required to achieve a difference in bone loss of approximately 0.28–0.30 mm.

Following the standard procedure, to detect possible differences between treatments, we accepted a risk of 5% and a statistical power of 95.

Considering the fact that a recent systematic review [23] on the difference of bone loss between crestal and subcrestal placements reported non-significant differences between the two positions, in this study, we decided to perform two-tailed hypothesis testing.

After all of these considerations and by applying the above specified formula, it was found that a minimum of 28 observation for each group would be required. 

After performing the randomization between treatments, 29 implants were placed at the crestal level, while 33 implants were placed at the subcrestal level.

Randomization was performed between three groups because we wanted to compare the internal and external connections. However, for the study reported in this article, bone loss in implants placed at the crestal and subcrestal levels was analyzed only at the conical internal connection. This decision, taken to avoid the risk of a possible interaction between connection and implant position, implies that the sample size is smaller and equal to 29 in internal connection crestal implants and 33 in internal connection subcrestal implants.

### 2.3. Types of Implants

**Figure 1:**
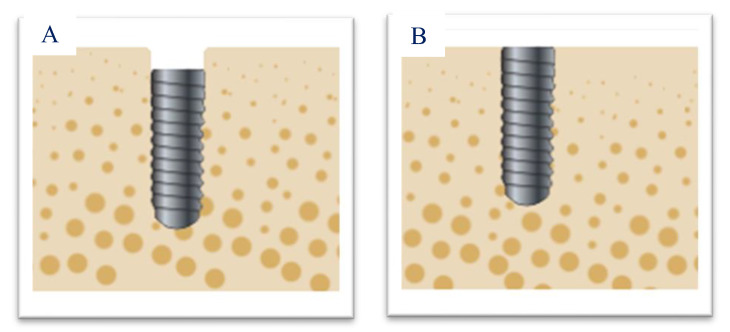
Images of implants placed at the subcrestal (**A**) and crestal (**B**) levels.

The implants belonged to ETK^®^ manufacturing (Barcelona, Spain), Naturactis^®^, and Naturall^®^. All implants were platform switching and had the same surface type: titanium oxide sandblasting and etching with nitric and hydrofluoric acid from the apex to the neck of the implant. The Naturactis^®^ implant, designed for subcrestal placement, consisted of the entire surface treated to promote bone formation on the entire surface of the implant neck. The Naturall^®^ implant designed for juxtacrestal positioning had a machined surface on the implant neck, with a micro-groove of 0.2 μm Ra for better mucosal adherence. (Figure 1).

The drilling sequence was carried out according to the instructions of the implant brand regarding bone quality. For placing all the implants, the motor was programmed at 32 rpm and 35 Ncm. 

### 2.4. Surgical Intervention and Clinical Follow-Up

Before surgery, all patients underwent a 0.2% chlorhexidine rinse (Bexident Post^®^ ISDIN SL., Barcelona, Spain); this protocol was carried out to reduce the bacterial load, since chlorhexidine, thanks to its bactericidal and bacteriostatic effects, would help us prevent the post-surgical infection [24].

The anesthesia used was 4% articaine with 1:100,000 epinephrine (Inibsa^®^, Lliça de Vall, Barcelona, Spain). We made a crestal incision and raised a full-thickness flap. The implants were placed by students in the 3rd year of the master’s degree in Medicine, Surgery, and Oral Implantology at the University of Barcelona, always with complete subordination. In this way, the same protocol was strictly followed, and we avoided the results obtained depending on the possible experience and/or preference of the operator. The entire procedure was supervised by NPG and JLL.

Regarding the healing period, in the implants placed in the mandible, 3 months were waited before performing the second phase and 4 months in the maxilla.

### 2.5. Radiographic Monitoring of Bone Loss

Standardized orthopantomography was performed. X-ray acquisition times were at the time of implant placement (T0), one month after implant placement (T1), and 3–4 months (T2). For better reproducibility, all measurements made on the radiographs were made by one examiner (NPG), and none of the implant placement surgeries were performed by the examiner (NPG). In addition, the patients were unaware of the position in which each type of implant was placed, so we can say that this is a single-blind study.

**Figure 2:**
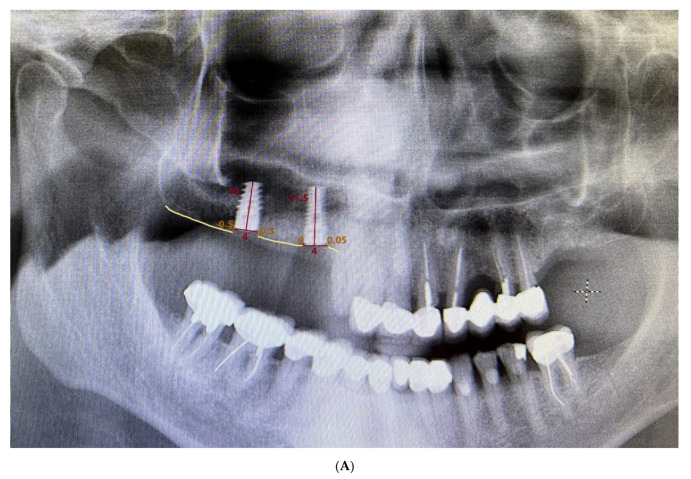

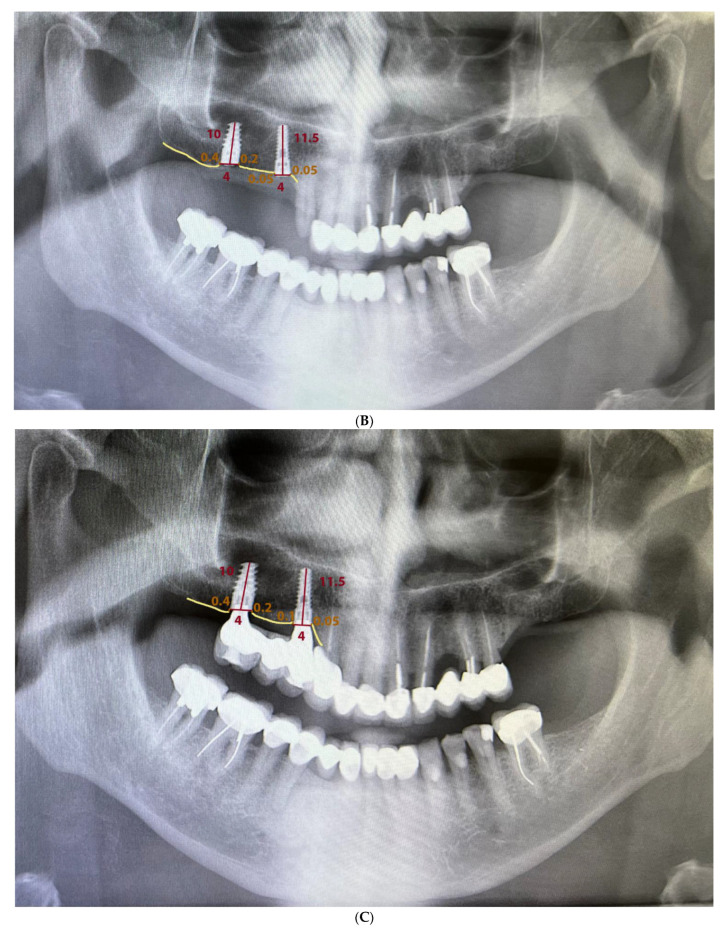
Measurement of peri-implant bone loss: (**A**) after implant placement, (**B**) after 1 month, and (**C**) after 4 months.

Measurements were made by using Planmeca Romexis^®^ software to process 2D images generated by the X-ray units. The calibration tool ensured that the measurement was correctly performed, given that it takes into account the length of each implant. Since all patients were required to have a CBCT before surgery, it was decided not to perform cone-beam radiographs to limit multiple radiation exposures. To carry out the measurements, two points were marked on the implant platform that, when joined horizontally, represented zero height. Perpendicular to this line, two straight lines were drawn mesial and distal to the implant until contact with the bone. If the implant was coronal to the bone, the result would be interpreted as negative, whereas, if the implant was infracrestal, the result would be positive. The difference between the mean of the mesial and distal measurements at different times was used to calculate the bone loss in the orthopantomography (Figure 2).

### 2.6. Resonance Frequency Analysis (RFA)

Implant stability measurements were performed by using RFA with the Penguin RFA^®^ device (Penguin RFA, Klockner Barcelona, Spain) in three moments. The measurement times were the day of implant placement, when the healing abutment was placed (second phase), and before the placement of the prosthesis. Two measurements were made for each implant: one vestibular–palatal from the buccal side and one mesiodistal from the mesial side. The recording times were decided according to the study carried out by Pozzi et al. in 2014 [25].

### 2.7. Statistical Analysis

The data collected were processed by using the statistical package STATA 14.0. A descriptive analysis of the qualitative and quantitative variables was performed.

The relationship between bone loss and implant position was analyzed by using the parametric *t*-test. The level of statistical significance chosen was 5% (α = 0.05).

To evaluate the association between the dependent variable, bone loss from T0 to T2, and the independent variables, placement C or placement S, a multiple linear regression analysis was used. All the variables that were considered to potentially affect the relationship of the study, such as sex, age, and smoker, were used as control variables in the regression, as well as the number of cigarettes per day, maxillary type, ISQ (implant stability quota), and mesial and distal implant contact.

## 3. Results

### 3.1. Patient Data

A total of 27 patients (12 women and 15 men) with an average age of 56 years, an average height of 1.67 m, and an average weight of 75 kg participated in this study.

There were no dropouts. Regarding brushing frequency, 19% of the patients reported brushing their teeth 2 or 3 times a day, 67% 1 or 2 times a day, and 15% not once a day. According to the classification of periodontal diseases taken from the World Workshop in 1989 [26], five patients had gingivitis associated with dental plaque, four patients had generalized chronic periodontitis, and one patient localized chronic periodontitis. Periodontitis is a disease that contributes to the loss of the supporting tissue of teeth and implants. Patients with generalized periodontitis would continue to undergo plaque control of the disease and, if necessary, a treatment based on the elimination of bacterial deposits that adhere to the surface of the tooth and the implant, accompanied by antimicrobial therapy [24]. The control visits would be carried out every 6 months in the periodontics service, where, in addition to the conventional treatment, in many cases, diode laser treatments are carried out, since it has been seen that they are very effective in reducing anaerobic microorganisms that inhabit the pockets [27]. A total of 26% were smokers (four women and three men), of whom two smoked more than 20 cigarettes a day, one patient between 11 and 15 cigarettes a day, three patients between 6 and 10, and one patient less than 5 cigarettes a day. A total of 89% had partial edentulism and 11% total edentulism (Table 1).

### 3.2. General Data of the Implants

Sixty-two implants were placed (33 designed for infracrestal placement and 29 crestal). During surgery, there were no complications such as fenestrations or dehiscences, and complete closure of the wound without tension was achieved in all cases.

One implant was mobile at the time of the second phase and was explanted, obtaining a survival rate of 98.4%. Most of the patients would be rehabilitated with single crowns and fixed prostheses. These prostheses were made by using CAD-CAM technology to ensure better passivity and control of distortion that can occur when performing procedures such as casting. Although monolithic zirconia would have been a favorable material due to its aesthetics, resistance, and less plaque accumulation, the material of choice in this study was conventional metal–ceramic [28].

In the case of totally edentulous patients, these would be rehabilitated with overdentures on Locators^®^ (Table 2).

### 3.3. Bone Loss

**Figure 3:**
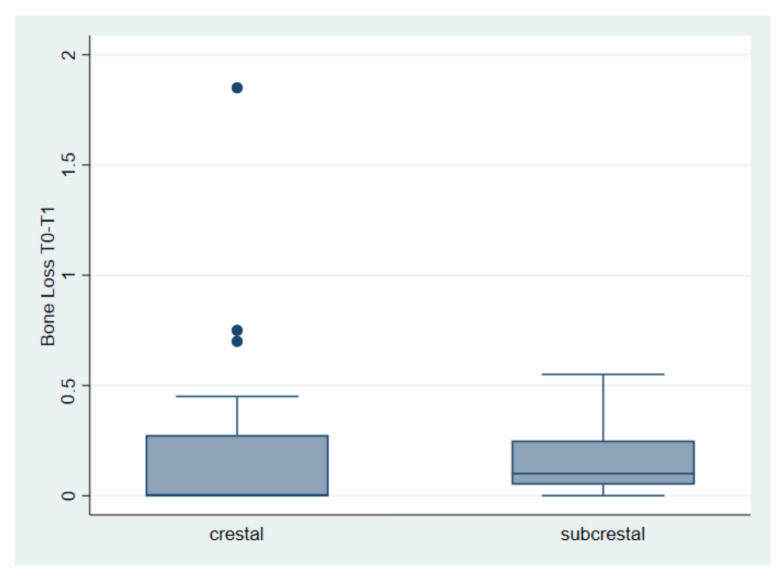
Bone loss (mm) in crestal vs. subcrestal implants between the first and second measurement (T0–T1). Bone loss of 0.19 mm for crestal implants and 0.15 mm for subcrestal implants.

The first comparison indicates an average bone loss of 0.19 mm for crestal implants and 0.15 mm for subcrestal implants. The said loss was found between the first and the second measurement. The test returned a *t* = 0.61, with a value of *p* = 0.54 (Table 3 and Figure 3).

**Figure 4:**
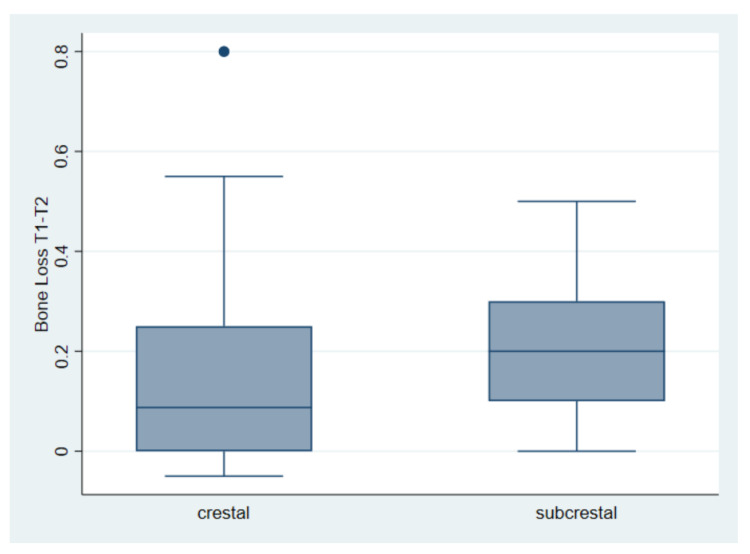
Bone loss (mm) in crestal vs. subcrestal implants between the second and third measurement (T1–T2). 0.17 mm for the crestal placement and 0.22 mm for the subcrestal placement.

The second comparison considers bone loss between the second and third measurements. The mean is 0.17 mm for the crestal placement and 0.22 mm for the subcrestal placement. The test returned us a t = −1.13, with a *p* = 0.26 (Table 4 and Figure 4).

**Figure 5:**
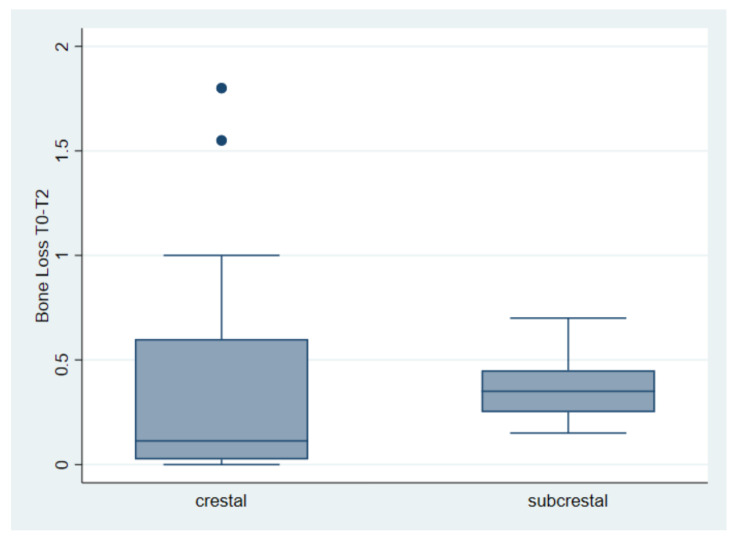
Bone loss (mm) in crestal vs. subcrestal implants between the first and third measurement (T0–T2). 0.359 and 0.367 mm, respectively, for the implant placed at the crestal and subcrestal level.

Finally, the third comparison takes into account the total bone loss from implant placement (T0) to the third measurement (T2), with an average loss of 0.359 and 0.367 mm, respectively, for the implant placed at the crestal and subcrestal level. The test returned a t = 0.07, with a value of *p* = 0.94 (Table 5, Figure 5). 

### 3.4. Resonance Frequency Analysis (RFA)

For each implant, an average of the vestibule–palatal and mesiodistal measurements was previously made. High ISQ values were obtained for all implants in the three measurement times performed.

The first measurement, the day of implant placement, refers to the primary stability obtained from the surgical act, and the following measurements, to the secondary stability obtained during the osseointegration process.

**Figure 6:**
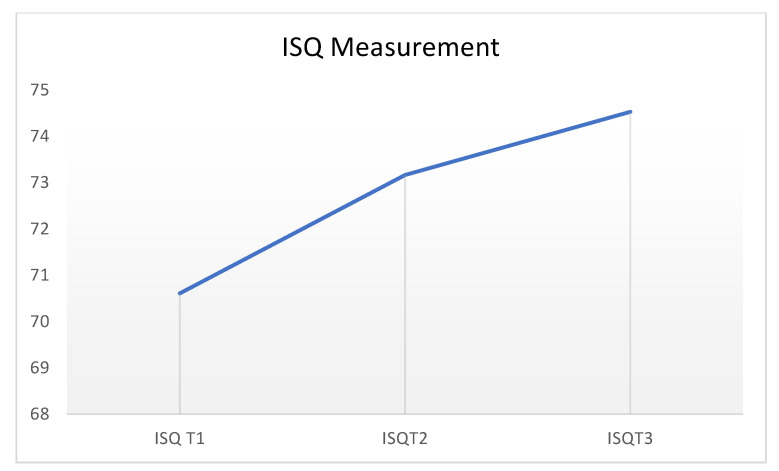
ISQ values at the three measurement times performed.

The measurements increased as the osseointegration period of the implants progressed (T0, 70.69; T1, 73.91; and T2, 75.32) (Table 6 and Figure 6).

The failed implant had a favorable ISQ on the day of placement. We did not record any additional failure of implants following the second ISQ measurement.

Table 7 reports the results of a multiple linear regression. The dependent variable (DV) is represented by the bone loss encountered between the first measurement (T0) and the last one (T2).

The treatment variable, i.e., the implant position, reports a positive although not statistically significant coefficient of 0.041, suggesting that the bone loss did not vary between the two positions. 

For the other possible determinants that could influence bone loss, no significant differences were found concerning age, sex, or tobacco consumption. The RFA of the implants in ISQ values did not give a statistically significant result regarding bone loss (Table 7). 

An R2 of 0.139 suggests that almost 14% of the variance was explained by the model. 

## 4. Discussion

This study evaluated the stability of the crestal bone after the implants were placed in different vertical positions. 

To reduce possible bias, we tried to control, as far as possible, all factors that, according to the current literature, can influence bone loss [22]. Among these factors we have the following: (1) For the type of connection, it was exactly the same in the two vertical positions. (2) For platform switching, the crest and subcrestal implant designs incorporated platform switching. (3) For prosthetic loading, all radiographic measurements of bone loss were performed prior to prosthetic function. (4) For the height of healing abutments, in this study, all implants received closure abutments that did not exceed the height or diameter of the implant platform. (5) For implant surfaces, it was established that, in order to deepen an implant, it must have a completely treated surface; otherwise, if the implant has a polished neck, the bone will not integrate, and necrosis of this bone could occur [29]. In our study, to avoid possible bias, the subcrestal implants had a full surface treatment, and the crestal implants had a polished neck.

Evidence suggests that both approaches to implant placement are clinically acceptable in terms of the peri-implant tissue parameters and survival of the implant-supported restoration [30].

Regarding the RFA analysis of the implants, it is considered that the values obtained were high in the three measurement times performed. A trend toward an increase in the average of the measurements could also be observed as the osseointegration period of the implants progressed.

The failed implant was detected on the day of the second phase, before the second ISQ measurement (T1). This implant had good primary stability with a high RFA value on the day of placement. This information suggests that, with respect to implant survival, the value gathered the day in which the implant was placed is not a key factor. In this study, no more implant failures were recorded after the second RFA measurement, so the values reached during the placement of the healing abutment could give us predictable information about the prognosis of the implant. These findings would coincide with the work of Rodrigo et al. (2010), who studied the relationship between the diagnosis of implant stability and its impact on implant survival, concluding that, contrary to the RFA values of primary stability, only secondary stability values were able to significantly predict the prognosis of implants [31].

The survival and success rate of the implants in this study (98.4%) is similar to the current survival rate described by Pjetursson et al. (2007), who reported that, in the last decade, the survival rate of implants has increased from 93.5 to 97.1% [32].

In the variable smoking and number of daily cigarettes, it is known that tobacco influences negatively, since it contributes to a greater number of implant failures, postoperative infections, and marginal bone loss than in the case of non-smoking patients [33]. In our study, smokers did not present greater bone loss compared to non-smokers. However, in this study, a vast majority of patients were non-smokers, and the follow-up time was short, so we cannot draw reliable conclusions.

In our study, no differences were found regarding bone loss and the type of maxilla. Over the years, it has been suggested that implants placed at the level of the mandible have higher survival rates than those of the maxilla. The basic cause of the difference in survival rates is considered to be the difference between bone qualities [34].

Currently, research efforts are directed toward understanding what causes bone loss, particularly during the first months after the placement of the implant. The results show that there are multiple factors that can cause bone loss that can be present individually or simultaneously and possibly interacting between them, and this fact makes it particularly tedious to disentangle the importance of each factor individually.

## 5. Conclusions

Considering the limitations of this study, it can be suggested that, in implants with the same surface, the two implant placement positions are clinically acceptable in terms of bone loss, clinical aspects, and survival. However, to assess whether bone loss differs significantly between the two vertical positions, further clinical trials with larger samples and longer follow-up periods are needed.

## Figures and Tables

**Table 1:** General patient data.

General Patient Data		Total
Sex	Women	12
	Male	15
Average age		56 years
Average height		1.67 m
Middle weight		75 kg
Number of cigarettes per day	1–5	1 patient
	6–10	3 patients
	11–15	1 patient
	16–20	0 patients
	≥20	2 patients
Smoker	No	20 patients; 74%
	Si	7 patients; 26%
Periodontal diseases	Gingivitis	5 patients
	Generalized chronic periodontitis	4 patients
	Localized chronic periodontitis	1 patient
Edentulism	Partial	24 patients 89%
	Total	3 patients 11%
Brushing frequency	0 times a day	4 patients 15%
	1 or 2 times a day	18 patients 67%
	3 or more times a day	5 patients 19%

**Table 2:** General implant data.

General Implant Data		Total
62 implants	Maxilla	40%
	Mandible	60%
At the molar level	50%	
At the level of premolars	34%	
At the incisor level	16%	
27 patients	24 patients	Crowns and bridges
	3 patients	Overdentures

**Table 3:** Bone loss in crestal and subcrestal implants.

	T0–T1		
	Average	SD	95% CI
C	0.19	0.39	(0.04–0.34)
S	0.15	0.14	(0.1–0.19)
*t*-test	0.61		
*p*-value	0.54		

C, crestal; S, subcrestal; SD, standard deviation; CI, confidence interval; T0, day of implant placement; T1, one month after placement.

**Table 4:** Bone loss in crestal and subcrestal implants.

	T1–T2		
	Average	SD	95% CI
C	0.17	0.20	(0.08–0.24)
S	0.22	0.14	(0.17–0.26)
*t*-test	−1.13		
*p*-value	0.26		

C, crestal; S, subcrestal; SD, standard deviation; CI, confidence interval; T1, one month after placement; T2, prosthetic restoration.

**Table 5:** Bone loss in crestal and subcrestal implants.

	T0–T2		
	Average	SD	95% CI
C	0.36	0.47	(0.17–0.54)
S	0.36.7	0.15	(0.31–0.42)
*t*-test	−0.07		
*p*-value	0.94		

C, crestal; S, subcrestal; SD, standard deviation; CI, confidence interval; T0, day of implant placement; T2, prosthetic restoration.

**Table 6:** ISQ (implant stability quota) values.

Measurement Times	Mean	ED	95% CI
**ISQ T0: Day of implant placement**	70.6065	9.67	(68.7–72.7)
**ISQ T1: Day of healing abutment placement (second stage)**	73.165	7.19	(72.4–75.4)
**ISQ T2: During the performance of the prosthetic restoration**	74.5275	6.15	(74–76.6)

**Table 7:** Determinants of bone loss between T0 and T2.

DV: Bone Loss between T0 and T2	
**Variable**	Coefficient
**Implant Position (Subcrestal)**	0.041
**Gender (Male)**	−0.001
**Age**	−0.011
**ISQ T2**	−0.003
**Smoker**	−0.081
**Constant**	1.219 *
**Prob > F**	0.146
**R2**	0.139

* 5% significance level; Constant, value at which the regression line crosses the *y*-axis; Prob, probability; F-value determines whether adding each of the independent variables improves the regression model; R2, proportion of the variance for a dependent variable explained by the independent variables.

## Data Availability

Not applicable.
